# Effect of exercise on bone health in children and adolescents with cancer during and after oncological treatment: A systematic review and meta-analysis

**DOI:** 10.3389/fphys.2023.1088740

**Published:** 2023-03-14

**Authors:** Andres Marmol-Perez, Esther Ubago-Guisado, Andrea Rodriguez-Solana, Jose J. Gil-Cosano, Vicente Martinez-Vizcaino, Ivan Cavero-Redondo, Jonatan R. Ruiz, Luis Gracia-Marco

**Affiliations:** ^1^ Department of Physical Education and Sports, Faculty of Sports Science, Sport and Health University Research Institute (iMUDS), University of Granada, Granada, Spain; ^2^ Instituto de Investigación Biosanitaria, ibs.Granada, Granada, Spain; ^3^ Department of Communication and Education, Universidad Loyola Andalucía, Sevilla, Spain; ^4^ Health and Social Research Center, Universidad de Castilla La Mancha, Cuenca, Spain; ^5^ Facultad de Ciencias de la Salud, Universidad Autónoma de Chile, Talca, Chile; ^6^ Centro de Investigación Biomédica en Red Fisiopatología de la Obesidad y Nutrición (CIBERobn), Instituto de Salud Carlos III, Madrid, Spain

**Keywords:** bone mineral density, dual-energy x-ray absorptiometry, impact-loading exercise, paediatrics, physical activity, weight-bearing exercise

## Abstract

**Background:** Although regular physical activity and exercise programs might improve bone health caused by oncological treatment and the disease itself, it remains unknown the pooled effect of exercise interventions following frequency, intensity, time and type prescriptions.

**Objective:** This systematic review and meta-analysis aimed to synthesise evidence regarding the effectiveness of exercise interventions on bone health in children and adolescents with cancer during and after oncological treatment.

**Methods:** A systematic search was conducted in the MEDLINE (*via* PubMed), Web of Science and Scopus databases from November 2021 to January 2022. Randomised controlled trials (RCTs) and non-RCTs reporting pre-post changes of the effectiveness of exercise interventions on DXA-measured bone parameters in young population (1–19 years) during or after oncological treatment were included. Pooled (ESs) and 95% confidence intervals (95%CIs) were calculated. The Preferred Reporting Items for Systematic Reviews and Meta-Analyses guidelines were followed.

**Results:** A total of eight trials with 341 participants were included. The meta-analyses did not reveal a statistically significant increase in whole body areal bone mineral density (ES = 0.10; 95%CI: −0.14, 0.34), lumbar spine (ES = 0.03; 95%CI: −0.21, 0.26) or femoral neck (ES = 0.10; 95%CI: −0.37, 0.56). Similarly, during the oncological treatment phase the ES was 0.04 (95%CI: −0.17, 0.25) and after the ES was 0.07 (95%CI: −0.20, 0.33).

**Conclusion:** To date, exercise interventions have been inappropriate and therefore, ineffective to illustrate any beneficial effect on bone health in children and adolescents with cancer during and after oncological treatment.

**Systematic Review Registration**: PROSPERO registration number: CRD42022310876

## Highlights

- Radiotherapy and chemotherapy, and cancer itself can affect bone mass between 20% and 50% of paediatric cancer patients through endocrine complications, such as gonadal dysfunction, growth hormone deficiency, and altered body composition.
- Exercise interventions to date have not been effective at improving bone health of children and adolescents with cancer during or after oncological treatment.
- There is a need of implementing well-designed exercise interventions in RCTs specifically focused on improving bone health in children and adolescents diagnosed with cancer.

## 1 Introduction

Paediatric cancer survival has experienced an unparalleled increase because of the advances in cancer detection and treatment (Miller et al., 2020). The current overall 5-year survival rate has risen up to 85% in children and adolescents (Trama et al., 2016; Siegel et al., 2023). However, all oncological treatments and the disease itself can decrease bone mass through endocrine alterations, such as gonadal dysfunction, growth hormone deficiency, and altered body composition (Marcucci et al., 2019). This is shown by a decreased bone formation and increased bone resorption in cancer-treated children (Kelly and Pottenger, 2022)*.* Research has shown that between 20% and 50% of paediatric cancer patients present impaired bone mass (Wilson and Ness, 2013; Marcucci et al., 2019). Moreover, paediatric cancer occurs during a critical phase for bone development and bone strengthening, since up to 95% of the adult bone mass may be accrued by the end of adolescence (Bailey et al., 1999; Harel et al., 2007; Rizzoli et al., 2010). Therefore, implementing feasible strategies to counteract cancer-related bone loss are vital to optimize skeletal health during growth and reduce the risk of osteoporosis later in life.

Although acquiring the peak bone mass strongly depends on genetics (Davies et al., 2005; Bonjour et al., 2007) regular physical activity and exercise programmes may contribute to achieve it (Weaver et al., 2016). Evidence has shown that exercise is safe during and after paediatric oncological treatment, even during the most aggressive phases (i.e., hematopoietic stem cell transplantation) (Campbell et al., 2019; Morales et al., 2021) and hence, it might contribute to preserve bone health in paediatric cancer patients during and after oncological treatment (Marcucci et al., 2019; Rodd et al., 2022). Weight-bearing impact exercise of high intensity including strains in different axes and multiple rest periods is known to improve bone mass (Ubago-Guisado et al., 2016; Ubago-Guisado et al., 2019). Interestingly, a systematic review showed that plyometric jump training causes improvements in areal bone mineral density (aBMD), bone mineral content (BMC) and structural properties in healthy children and adolescents (Gómez-Bruton et al., 2017). In adolescent males, a jump-based intervention enhanced bone parameters in those engaged in non-osteogenic sports and with poorer bone health (Vlachopoulos et al., 2018a; Vlachopoulos et al., 2018b). However, there is limited evidence of the effects of exercise on bone parameters in paediatric cancer patients, the reported findings are inconsistent (Hartman et al., 2009; Waked and Albenasy, 2018) and some of the studies have been carried out in a very small sample of participants (Müller et al., 2014; Dubnov-Raz et al., 2015). The interest in exercise oncology has sharply risen during the last decade and therefore, there is a need to know the pooled effect of exercise interventions on bone health in young paediatric cancer patients.

Therefore, the aims of this systematic review and meta-analysis were to (i) determine the pooled effect of exercise interventions from randomised controlled trials (RCTs) and non-RCTs in children and adolescents with cancer during and after oncological treatment on bone health and (ii) explore factors influencing the response of the exercise intervention. We hypothesised that (i) exercise would have a positive effect on bone health in this population when compared with control non-exercise groups, and (ii) enhancements will be greater in studies with longer interventions that involve weight-bearing and impact exercises of high intensity.

## 2 Methods

This study was conducted according to the Preferred Reporting Items for Systematic Reviews and Meta-Analyses (PRISMA) guidelines (Supplementary Appendix S1) (Page et al., 2021; Ardern et al., 2022).

### 2.1 Search strategy

This systematic review and meta-analysis were registered in the International Prospective Register for Systematic Reviews (PROSPERO; registration number CRD42022310876). The recommendations of the Cochrane Collaboration Handbook for conducting systematic reviews and meta-analyses were strictly followed (Higgins et al., 2022). A systematic search of the literature was conducted in various electronic databases: MEDLINE (*via* PubMed), Web of Science and Scopus databases from November 2021 to January 2022. Intervention studies addressing the change in aBMD and BMC after exercise programmes in paediatric cancer participants in the childhood and adolescence periods were eligible. This systematic search was only restricted by language, solely including those studies published in English. We also manually screened other sources for additional records (i.e., references from previous reviews) and contacted authors for missing information when necessary. No studies were included from manual screenings. Combinations of the following keywords were used in the search (Supplementary Appendix S2): exercis*, move*, moving, sport*, train*, “physical activity”, weightbear*, “high impact”, running, walk*, strength*, “physical fitness”, step*, gymnastic, balance, bone, cancer, onco*, myelo*, leukaemia, leukemia, neoplasm*, lympho*, carcinoma, tumor, tumour, sarcoma, child*, adolescen*, young*, boy*, girl*, pediatric*, paediatric*, trial*, random, intervention*, program* and rehabilitation. The literature search was complemented by reviewing references of the articles considered eligible.

### 2.2 Study selection

**FIGURE 1:**
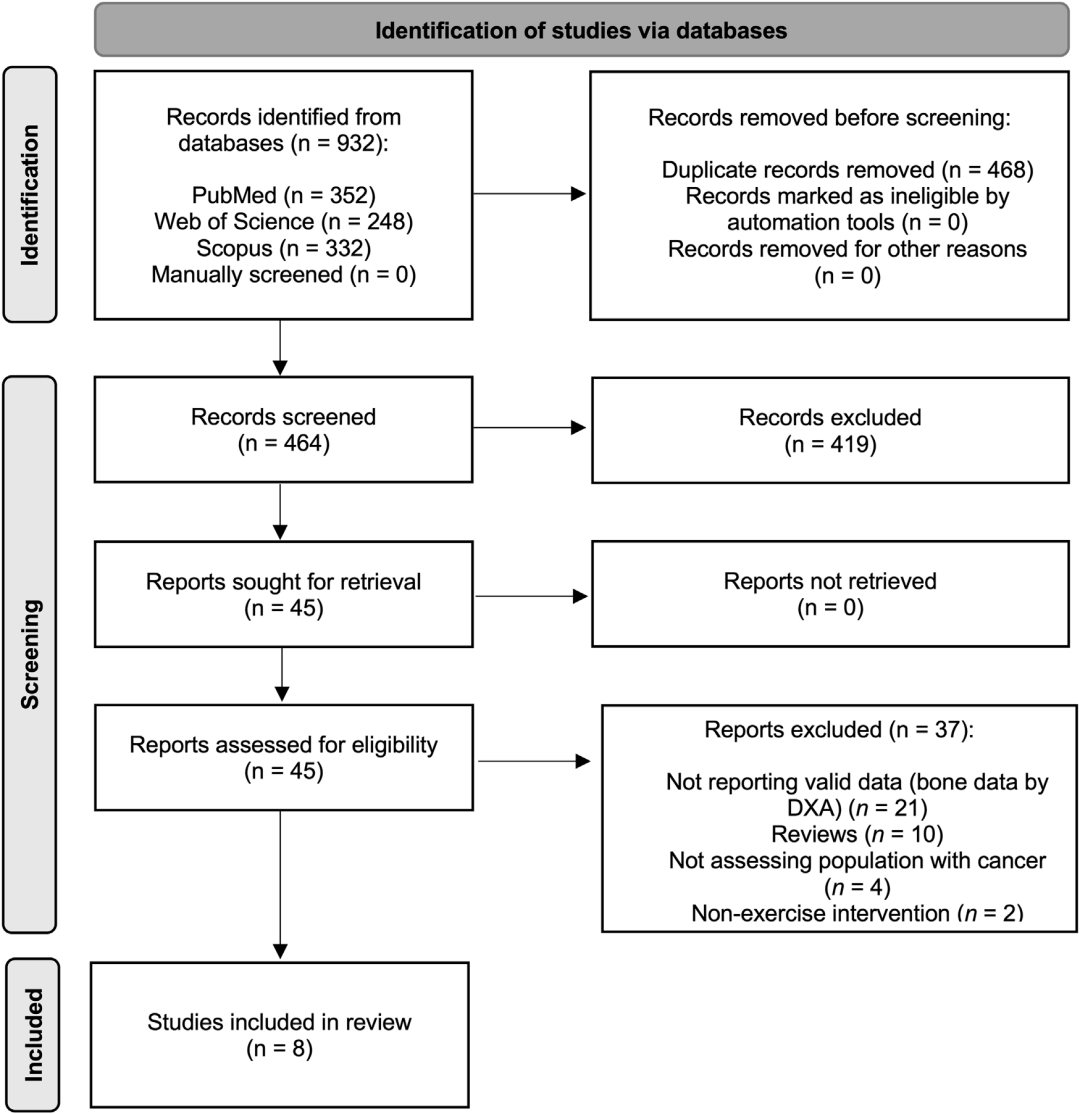
Literature search Preferred Reporting Items for Systematic Reviews and Meta-Analyses (PRISMA) consort diagram.

Study inclusion criteria were as follows: (i) participants: Paediatric cancer population (aged 1–19 years) during and after oncological treatment irrespective of the type of the treatment at any time point; (ii) study design: Intervention studies based on exercise programmes (RCT and non-RCTs) with a non-exercising control group; (iii) exposure: Exercise programmes with a minimum of 1 month of duration without restrictions on the setting, resistance, aerobic, walking, gymnastic, yoga, whole-body vibration and balance interventions were included, no minimal adherence required and the concomitant exposure to other treatment such as nutritional supplementation with calcium or vitamin D to both groups was allowed; and (iv) outcome: aBMD and BMC assessed using dual-energy X-ray absorptiometry (DXA). Exclusion criteria were as follows: (i) studies including individuals older than 19 years old; (ii) non-eligible publication types, such as review articles, editorials, comments, guidelines or case reports; (iii) assessment of aBMD and BMC using other methods (i.e., computed tomography); and (iv) studies published in any language other than English. Based on the selection criteria, all studies were independently screened for inclusion by two reviewers and disagreements were solved by consensus or involving a third researcher. A total of potential manuscripts were identified following database examination (Figure 1), eight of them met the inclusion criteria and were, therefore, included in the meta-analysis.

### 2.3 Data extraction and risk of bias assessment

All articles retrieved from the respective databases were exported and handled in an EndNote library (Endnote version X7). After removing the duplicated articles, two researchers independently read the titles and abstracts to screened out the irrelevant articles according to the inclusion/exclusion criteria, and finally screened the articles by reading the full text. Any conflicts were solved by consensus with a third researcher.

The following data were retrieved from the original reports: (i) first author and year of publication; (ii) country from which the data were collected; (iii) study design; (iv) sample characteristics (age, sample size, body mass index, height, weight, and type of cancer); and (v) the method used for measuring bone measurement characteristics (aBMD, volumetric BMD, bone mineral apparent density and BMC, including values for whole body, lumbar spine, and femoral neck) at baseline and at end of follow-up. Besides, data concerning exercise programmes were extracted from the original manuscripts: (i) frequency, (ii) intensity, (iii) time, (iv) type, (v) volume, (vi) progression, (vii) intervention duration, (viii) attendance, (ix) supervision, (x) home exercise programmes, (xi) control group and (xii) other characteristics.

Two researchers independently assessed the risk of bias and any discrepancies were resolved by a third reviewer. The Cochrane Collaboration’s tool for assessing the risk of bias (RoB2.0) was used to assess the certainty of the evidence of the RCT studies (Sterne et al., 2019). This tool covers bias in five domains: Randomisation process, deviations from intended interventions, missing outcome data, measurement of the outcome, and selection of the reported result. According to this assessment tool, the studies were rated as “low risk of bias” (if all domains were judged as “low risk”), “some concerns” (if there was at least one domain rated as having “some concerns”), or “high risk of bias” (if there was at least one domain judged as “high risk”). The Joanna Briggs Institute Critical Appraisal Tool (Tufanaru et al., 2017) for Quasi-Experimental Studies were used to assess the certainty of the evidence of the non-randomised experimental studies. According to this assessment tool, the studies were rated as good (i.e., most criteria met, with a low risk of bias), fair (i.e., some criteria met, with a moderate risk of bias), or poor (i.e., few criteria met, with a high risk of bias). No studies were excluded based on the quality appraisal.

### 2.4 Statistical considerations

The inverse-variance-weighted method used to compute the pooled effect size (ES) estimate and the 132 respective 95% confidence interval (95%CI). An ES was calculated for the pre-post aBMD mean values or the mean value change using Sn’s d index. ES values of 0.2 were considered a weak effect, values of 0.5 were considered a moderate effect, values of 0.8 were considered a strong effect, and values larger than 1.0 were considered a very strong effect. When studies reported means and standard errors (SE) or 95% CI we used the formulas, standard deviation (SD) = sqrt (sample size) * SE and SD = sqrt (sample size) * [(upper limit 95% CI—lower limit 95% CI)/3.92], to convert to SD. Additionally, when studies reported pre-post aBMD mean values or the mean value change data in graphs, the online tool WebPlotDigitizer (https://apps.automeris.io/wpd/) was used to extract the data for the ES calculation.

Heterogeneity of results across studies was assessed using the I^2^ statistic (Higgins and Thompson, 2002). I^2^ values were considered as follows: might not be important (0%–40%), may represent moderate heterogeneity (30%–60%), may represent substantial heterogeneity (50%–90%), or considerable heterogeneity (75%–100%); the corresponding *p* values also were considered. Finally, we calculated the statistic τ2 to establish the size and clinical relevance of heterogeneity. A τ2 estimate of 0.04 can be considered as low, 0.14 as moderate, and 0.40 as a substantial degree of the clinical relevance of heterogeneity (Stettler et al., 2008).

Exploratory subgroups analyses were conducted according to the type of aBMD region (whole body, lumbar spine or femoral neck) and patient status (during oncological treatment or surviving patients). Furthermore, sensitivity analyses (systematic reanalysis while removing studies one at a time) and subgroup analyses were conducted to assess the robustness of the summary estimates. The results of the sensitivity analyses were considered meaningful when the resulting estimates were modified beyond the CIs of the original summary estimate. In addition, sensitivity analyses provided insight into whether any study or special condition included in the studies accounted for a large proportion of the heterogeneity among the ES pooled estimations, based on the change in I^2^ values (and associated categories previously reported).

Finally, small-study effects and publication bias were examined using the Doi plot and the Luis Furuya–Kanamori index (LFK index). No asymmetry, minor asymmetry or major asymmetry were considered with values of one, between one and two, and two, respectively (Furuya-Kanamori et al., 2018). Statistical analyses were performed using STATA SE software, version 14 (StataCorp, College Station, Texas).

## 3 Results

The PRISMA flow diagram for the systematic search and study selection is shown in Figure 1.

### 3.1 Level of evidence and risk of bias of the studies

The overall risk of bias for RCTs showed two studies with low risk (40%) and three studies with some concerns (60%) (Supplementary Appendix S3). Regarding the specific domains, in the randomisation process, missing outcome data, and measurement of outcome domains, all the studies (n = 5, 100%) were scored as low risk. In the deviations from intentional interventions and selection of the reported results domains, two studies (40%) were scored as some concerns and three studies as low risk (60%).

The risk of bias for non-randomised experimental studies showed two studies with high quality (66,67%) and one study with medium quality (33,33%). When the studies were analysed by individual domains, all the studies (n = 3, 100%) made clear what the ‘cause’ was and what the ‘effect’ was, had a control group, had multiple measurements of the outcome both pre and post the intervention/exposure, adequately described and analysed any differences between groups in terms of their follow up, and measured in the same way the outcomes of included participants and in a reliable way. In addition, two studies included similar participants (66.67%), and one study (33.3%) included participants in any comparisons receiving similar treatment/care other than the exposure or intervention of interest, and used an appropriate statistical analysis (Supplementary Appendix S4).

### 3.2 Characteristics of the participants and assessment methods selected

Table 1 shows the participants characteristics of the eight studies included in this meta-analysis. Participants age ranged from 1.3 to 18 years old, with sample sizes ranging from 21 to 75 participants (mean = 48 participants, total = 341). The type of cancer included acute lymphoblastic leukaemia, acute myeloid leukaemia, Hodgkin lymphoma or non-Hodgkin lymphoma, chronic myeloid leukaemia or Burkitt, central nervous system/brain tumour, solid tumour, neuroblastoma, Wiskott–Aldrich syndrome or osteosarcomas and Ewing sarcoma. Concerning the assessment methods carried out in the studies, five studies used the Lunar Prodigy, one study used the Hologic, one study used both the Lunar Prodigy or the Hologic and one study used both the Hologic and Lunar Prodigy.

**TABLE 1:** Descriptive characteristics of included studies.

			Population characteristics at baseline								Outcomes		
Reference	Country	Design	Age, years	Sample size [n (% male)]	BMI, kg/m^2^	Height, cm/m	Weight, kg	Cancer-type	Treatment phase/Type	Method		Baseline bone	Follow-up
Hartman et al. (2009)	Netherlands	Prospective randomised study	**Exercise group: Mean (range)** 5.3 (1.3–15.6)**Control group: Mean (range)** 6.2 (1.7–17.1)	**Exercise group:** 20 (56%)**Control group:**21 (62%)	**Exercise group: SDS**-0.33**Control group: SDS**-0.38	**Exercise group: SDS**-0.11**Control group: SDS**-0.10	**Exercise group: SDS**-0.40**Control group: SDS**-0.09	**Exercise group**: 25 Acute lymphoblastic leukemia**Control group**: 26 Acute lymphoblastic leukemia	During treatment/Chemotherapy	X-ray absorptiometry (DXA; Lunar DPX-L, Madison, WI)		**Exercise group: SDS** **WB aBMD** (g/cm^2^): −0.10**LS aBMD** (g/cm^2^): −0.42**LS BMAD** (g/cm^2^): 0.14**Control group: SDS** **WB aBMD** (g/cm^2^): −0.18**LS aBMD** (g/cm^2^): −0.96**LS BMAD** (g/cm^2^): −0.48	**Exercise group: SDS** **WB ΔaBMD** (g/cm^2^): 0.42**LS ΔaBMD** (g/cm^2^): 0.10**LS ΔBMAD** (g/cm^2^): 0.12**Control group: SDS** **WB ΔaBMD** (g/cm^2^): 0.35**LS ΔaBMD** (g/cm^2^): 0.14**LS ΔBMAD** (g/cm^2^): −0.04
Müller et al. (2014)	Germany	Non-randomised interventional study	**Exercise group:** **Mean ± SD** 15.2 ± 2.0 **Control group:** **Mean ± SD** 12.2 ± 2.6	**Exercise group:** 10 (40%) **Control group:** 11 (45%)	**Exercise group:** **Mean ± SD** 19.9 ± 2.9 **Control group:** **Mean ± SD** 18.2 ± 3.9	**Exercise group:** **Mean ± SD** 1.71 ± 0.10 **Control group:** **Mean ± SD** 1.54 ± 0.08	**Exercise group:** **Mean ± SD** 57.9 ± 7.2 **Control group:** **Mean ± SD** 44.0 ± 12.6	**Exercise group**: 7 osteosarcomas and 3 Ewing sarcoma **Control group**: 7 osteosarcomas and 4 Ewing sarcoma	During treatment/Surgery and/or radiotherapy	X-ray absorptiometry (DXA), Lunar Prodigy system (enCore 2006; Software version 10.51.006, GE Healthcare)		**Exercise group:** **Mean (SEM)** **LS (L2-L4) vBMD** (g/cm^3^): 0.348, (0.020) **LS (L2-L4) aBMD** (g/cm^2^): 1.074 (0.054) **LS (L2-L4) BMC** (g): 42.06 (2.58) **FN vBMD** (g/cm^3^): 0.418 (0.024) **FN aBMD** (g/cm^2^): 1.103 (0.043) **FN BMC** (g): 4.85 (0.27) **Control group:** **Mean (SEM)** **LS (L2-L4) vBMD** (g/cm^3^): 0.322 (0.019) **LS (L2-L4) aBMD** (g/cm^2^): 0.961 (0.050) **LS (L2-L4) BMC** (g): 35.47 (2.43) **FN vBMD** (g/cm^3^): 0.381 (0.023) **FN aBMD** (g/cm^2^): 0.898 (0.040) **FN BMC** (g): 4.06 (0.25)	**Exercise group: Mean (SEM)** **LS (L2-L4) vBMD** (g/cm^3^): 0.347, (0.018) **LS (L2-L4) aBMD** (g/cm^2^): 1.068 (0.055) **LS (L2-L4) BMC** (g): 41.23 (2.76) **FN vBMD** (g/cm^3^): 0.406 (0.027) **FN aBMD** (g/cm^2^): 0.998 (0.052) **FN BMC** (g): 4.72 (0.30) **Control group:** **Mean (SEM)** **LS (L2-L4) vBMD** (g/cm^3^): 0.294 (0.017) **LS (L2-L4) aBMD** (g/cm^2^): 0.875 (0.051) **LS (L2-L4) BMC** (g): 32.97 (2.60) **FN vBMD** (g/cm^3^): 0.332 (0.027) **FN aBMD** (g/cm^2^): 0.791 (0.052) **FN BMC** (g): 3.63 (0.30)
Cox et al. (2018)	United States and Canada	RCT	NR	**Exercise group:** 35 ( 64.2%) **Control group:** 40 (66.7%)	NR	NR	NR	**Exercise group**: 53 Acute lymphoblastic leukemia Control group: 55 Acute lymphoblastic leukemia	During treatment/Chemotherapy	Dual-energy X-ray absorptiometry (DEXA) using the GE Lunar Prodigy (Atlanta and Toronto) or the Hologic (SJCRH and MDA)		**Exercise group:** **LS (L1-L4):** **Z-score (SEM)** −0.21 (±1.27) ****Control group**:** **LS (L1-L4) Z-score:** **Z-score (SEM)** −0.62 (±1.14)	**Exercise group:** **LS (L1-L4) Z-score:** **Z-score (SEM)** **−**0.55 (±0.86) **Control group:** **LS (L1-L4) Z-score**: **Z-score (SEM)** −0.78 (±1.11)
Waked and Albenasy (2018)	Saudi Arabia	RCT	**Exercise group:** **Mean ± SD** 9.26 ± 2.39 **Control group:** **Mean ± SD** 9.91 ± 2.09	**Exercise group:** 23 (65.2%) **Control group:** 23 (78.3%)	**Exercise group:** **Mean ± SD** 18.15 ± 1.79 **Control group:** **Mean ± SD** 19.12 ± 1.56	**Exercise group:** **Mean ± SD** 124.13 ± 11.95 **Control group:** **Mean ± SD** 129.30 ± 10.8	**Exercise group:** **Mean ± SD** 28.52 ± 7.39 **Control group:** **Mean ± SD** 32.26 ± 6.57	**Exercise group**: 23 Acute lymphoblastic leukemia **Control group**: 23 Acute lymphoblastic leukemia	During treatment/Chemotherapy	Dual Energy X-ray Absorptiometry (DEXA) (DXA, Lunar DPXL/PED, Madison, Wisconsin, United States).		**Exercise group:** **Mean (SD)** **WB aBMD** (g/cm^2^): 0.811 ± 0.072 **LS (L2-L4) aBMD** (g/cm^2^): 0.727 ± 0.059 **Control group:** **Mean (SD)** **WB aBMD** (g/cm^2^): 0.814 ± 0.071 **LS (L2-L4) aBMD** (g/cm^2^): 0.712 ± 0.050	**Exercise group:** **Mean (SD)** **6** **months** **WB aBMD** (g/cm^2^): 0.842 ± 0.076 **LS (L2-L4) aBMD** (g/cm^2^): 0.778 ± 0.035 **12** **months** **WB aBMD** (g/cm^2^): 0.869 ± 0.069 **LS (L2-L4) aBMD** (g/cm^2^): 0.808 ± 0.058 **Control group:** **Mean (SD)** **6** **months** **WB aBMD** (g/cm^2^): 0.805 ± 0.056 **LS (L2-L4) aBMD** (g/cm^2^): 0.716 ± 0.040 **12** **months** **WB aBMD** (g/cm^2^): 0.797 ± 0.055 **LS (L2-L4) aBMD** (g/cm^2^): 0.724 ± 0.032
Dubnov-Raz et al. (2015)	Israel	Interventional trial	**Exercise group:** **Mean (range)** 11.1 (7.8–13.8) **Control group:** **Mean (range)** 11.8 (9.0–12.8)	**Exercise group:** 10 (40%) **Control group:** 11 (50%)	**Exercise group:** **Mean (range)** 19.6 (17.6–3.9) **Control group:** **Mean (range)** 18.7 (17.1–21.2)	**Exercise group:** **Mean (range)** 144 (130–152) **Control group:** **Mean (range)** 148 (127–158)	**Exercise group:** **Mean (range)** (33.0–52.8) **Control group:** **Mean (range)** 40.6 (29.1–54.9)	**Exercise group**: 5 Acute lymphoblastic leukemia, 1 Burkitt lymphoma, 1 acute myeloid leukemia, 1 acute promyelocytic leukemia, 1 juvenile myelomonocytic leukemia and 1 neuroblastoma **Control group**: 3 Acute lymphoblastic leukemia, 2 Burkitt lymphoma, 2 Hodgkin lymphoma, 1 medulloblastoma, 1 rhabdomyosarcoma, 1 Wilms’ tumor, 1 severe aplastic anemia and 1 Wiskott–Aldrich syndrome	After treatment/Chemotherapy and/or steroids and/or bone marrow transplantation	Dual energy X-ray absorptiometry with Lunar DPX software version 3.6 (Lunar Prodigy; General Electric Healthcare, Madison, Wisconsin, United States)		**Exercise group: Median (IQR)** **B aBMD** (g/cm^2^): 0.95 (0.87–1.01) **WB BMC:** (g): 1435 (1117–2051) **LS (L1-L4) aBMD** (g/cm^2^): 0.84 (0.78–0.92) **FN aBMD** (g/cm^2^): 0.85 (0.75–0.89) **Control group: Median (IQR)** **WB aBMD** (g/cm^2^): 0.90 (0.87–0.99) **WB BMC** (g): 1293 (1124–2069) **LS (L1-L4) aBMD** (g/cm^2^): 0.75 (0.63–0.82) **FN aBMD** (g/cm^2^): 0.82 (0.70–0.97)	**Exercise group:** **Median (IQR)** **WB aBMD** (g/cm^2^): 0.97 (0.86–1.03) **WB BMC** (g): 1631 (1076–1993) **LS (L1-L4) aBMD** (g/cm^2^): 0.88 (0.79–0.97) **FN aBMD** (g/cm^2^): 0.89 (0.82–0.95) **Control group:** **Median (IQR)** **WB aBMD** (g/cm^2^): 0.91 (0.90–1.03) **WB BMC** (g): 1445 (1222–2139) **LS (L1-L4) aBMD** (g/cm^2^): 0.79 (0.69–0.85) **FN aBMD** (g/cm^2^): 0.86 (0.72–0.97)
Braam et al. (2018)	Netherlands	RCT	**Exercise group:** **Mean ± SD** 13.4 ± 3.1 **Control group:** **Mean ± SD** 13.1 ± 3.1	**Exercise group:** 26 (53%) **Control group:** 33 (55%)	NR	**Exercise group:** **Mean ± SD** 158.9 ± 16.5 **Control group:** **Mean ± SD** 154.5 ± 17.2	**Exercise group:** **Mean ± SD** 51.6 ± 16.0 **Control group:** **Mean ± SD** 49.2 ± 16.9	**Exercise group**: 8 Acute lymphoblastic leukemia, 12 acute myeloid leukemia or Hodgkin lymphoma or non-Hodgkin lymphoma or chronic myeloid leukemia or Burkitt, 1 central nervous system/brain tumor and 9 solid tumors **Control group**: 12 Acute lymphoblastic leukemia, 13 acute myeloid leukemia or Hodgkin lymphoma or non-Hodgkin lymphoma or chronic myeloid leukemia or Burkitt, 6 centrals nervous system/brain tumor and 7 solid tumors	After treatment/Chemotherapy and/or radiotherapy	Dual-energy-X-ray absorptiometry (DXA)-scanner. (Hologic DXA scanner with the same software) + Lunar		**Exercise group:** **Mean (SD)** **LS (L1-L4) aBMD** (g/cm^2^): 0.78 (±0.21) **Control group:** **Mean (SD)** **LS (L1-L4) aBMD** (g/cm^2^): 0.75 (±0.18)	**Exercise group:** **Mean (SD)** Post Short-term **LS (L1-L4) aBMD** (g/cm^2^): 0.78 (±0.20) Post Long-term **LS (L1-L4) aBMD** (g/cm^2^): 0.83 (±0.23) **Control group:** **Mean (SD)** Post Short-term **LS (L1-L4) aBMD** (g/cm^2^): 0.76 (±0.20) Post Long-term **LS (L1-L4) aBMD** (g/cm^2^): 0.78 (±0.21)
Mogil et al. (2016)	United States	Prospective, double-blind, placebo-controlled trial	**Exercise group:** **Mean ± SD** 13.6 ± 3.7 **Control group:** **Mean ± SD** 13.6 ± 2.9	**Exercise group:** 22 (56.2%) **Control group:** 26 (51.5%)	NR	NR	NR	NR	After treatment/Unspecified	X-ray absorptiometry (DEXA, 4500 QDR-A/Discovery fan beam; Hologic		NR	**Exercise group:** **Mean change (SD)** **WB BMC/height, total, %:** 1.71 (9.01) **WB BMD/height, total, %:** 6.56 (7.64) **LS BMC/height, total, %:** 3.70 (21.20) **LS BMD/height, total, %:** 4.91 (10.34) **LS vBMD, %:** 5.64 (10.83) **Control group:** **Mean change (SD)** **WB BMC/height, total, %:** 3.99 (8.97) **WB BMD/height, total, %:** 3.45 (7.60) **LS BMC/height, total, %:** 2.54 (21.06) **LS BMD/height, total, %:** 5.01 (10.29) **LS vBMD, %:** 5.30 (11.06)
Elnaggar and Mohamed (2021)	Saudi Arabia	Prospective, single-blinded quasi-experimental study	**Exercise group:** **Mean ± SD** 13.33 ± 3.13 **Control group:** **Mean ± SD** 12.87 ± 2.56	**Exercise group: 15 (73.3%) Control group: 15 (53.3%)**	**Exercise group:** **Mean ± SD** 22.53 ± 1.40 **Control group:** **Mean ± SD** 21.89 ± 1.57	**Exercise group:** **Mean ± SD** 145 ± 14 **Control group:** **Mean ± SD** 149 ± 0.13	**Exercise group:** **Mean ± SD** 48.20 ± 10.86 **Control group:** **Mean ± SD** 49.80 ± 11.54	**Exercise group**: 15 Acute lymphoblastic leukemia Control group: 15 Acute lymphoblastic leukemia	After treatment/Unspecified	Lunar DPX-L pediatric software and dual-energy x-ray absorptiometry (DEXA) device (GE-Lunar)		**Exercise group:** **Mean (SD)** **LS (L**_**2**_ **through L**_**5**_ **segment) aBMD** (g/cm^2^): 0.64 ± 0.10 **LS (L**_**2**_ **through L**_**5**_ **segment) vBMD** (g/cm^3^): 0.32 ± 0.04 **LS BMC (L**_**2**_ **through L**_**5**_ **segment)** (g): 33.91 ± 7.12 **FN aBMD** (g/cm^2^): 0.59 ± 0.06 **FN vBMD** (g/cm^3^): 0.31 ± 0.04 **FN BMC** (g): 32.35 ± 6.69 **Control group:** **Mean (SD)** **LS (L**_**2**_ **through L**_**5**_ **segment) aBMD** (g/cm^2^): 0.61 ± 0.06 **LS (L**_**2**_ **through L**_**5**_ **segment) vBMD** (g/cm^3^): 0.30 ± 0.03 **LS (L**_**2**_ **through L**_**5**_ **segment) BMC** (g/cm): 30.63 ± 5.92 **FC aBMD** (g/cm^2^): 0.62 ± 0.05 **FN vBMD** (g/cm^3^): 0.30 ± 0.04 **FN BMC** (g): 32.88 ± 6.16	**Exercise group:** **Mean (SD)** **LS (L**_**2**_ **through L**_**5**_ **segment) aBMD** (g/cm^2^): 0.70 ± 0.06 **LS (L**_**2**_ **through L**_**5**_ **segment) vBMD** (g/cm^3^): 0.36 ± 0.03 **LS (L**_**2**_ **through L**_**5**_ **segment) BMC** (g): 37.46 ± 4.59 **FN aBMD** (g/cm^2^): 0.67 ± 0.07 **FN vBMD** (g/cm^3^): 0.34 ± 0.03 **FN BMC** (g): 37.76 ± 5.65 **Control group:** **Mean (SD)** **LS (L**_**2**_ **through L**_**5**_ **segment) aBMD** (g/cm^2^): 0.64 ± 0.07 **LS (L**_**2**_ **through L**_**5**_ **segment) vBMD** (g/cm^3^): 0.32 ± 0.03 **LS (L**_**2**_ **through L**_**5**_ **segment) BMC** (g): 33.29 ± 4.14 **FC aBMD** (g/cm^2^): 0.63 ± 0.05 **FN vBMD** (g/cm^3^): 0.31 ± 0.04 **FN BMC** (g): 34.45 ± 5.02

Abbreviations: RCT, randomised controlled trial; WB, whole body; LS, lumbar spine; FN, femoral neck; aBMD, areal bone mineral density; vBMD, volumetric bone mineral density; BMAD, bone mineral apparent density; BMC, bone mineral content; SDS, standard deviation scores; NR, not reported.

### 3.3 Characteristics of the studies selected

These eight studies reported aBMD and BMC changes after exercise interventions in paediatric cancer survivors during (n = 4) and after (n = 4) oncological treatment (Table 1) (Hartman et al., 2009; Müller et al., 2014; Dubnov-Raz et al., 2015; Mogil et al., 2016; Braam et al., 2018; Cox et al., 2018; Waked and Albenasy, 2018; Elnaggar and Mohamed, 2021), compared with a non-exercising control group. They were published between 2009 and 2021 and were carried out in six different countries: two studies conducted in Netherlands, two in Saudi Arabia, one in United States, one in United States and Canada, one in Israel and one in Germany.

There were five RCTs (Hartman et al., 2009; Mogil et al., 2016; Braam et al., 2018; Cox et al., 2018; Waked and Albenasy, 2018) and 3 non-RCTs (Müller et al., 2014; Dubnov-Raz et al., 2015; Elnaggar and Mohamed, 2021). Table 2 shows the eight screened studies highlighting their FITT interventions. The characteristics of the interventions are as follows: (i) Frequency, ranged 1 from 1.5 to 7 days a week (mean = 3 days a week); (ii) Intensity, was differently reported depending on the type of exercise in terms of heart rate peak (HRpeak), mechanical stimulation from a platform, Borg’s scale, weight-bearing, light-to-moderate, moderate-to-vigorous and high intensity, while two studies did no describe the intensity target; (iii) Time per session, ranged from 10 to 60 min (mean = 36 min) but one study did not report it and time per intervention, ranged from 3 to 30 months (mean = 13 months); and (iv) Type, four studies conducted a concurrent exercise intervention (resistance and endurance training), three studies implemented a resistance training intervention and one study carried out a low-magnitude, high-frequency mechanical stimulation. Control groups did not receive an exercising treatment.

**TABLE 2:** Intervention characteristics of included studies.

Reference	Frequency (F)	Volume (V)	Supervision (S)
	Intensity (I)	Progression (P)	Place of exercise program (PEP)
	Time (TM)	Intervention duration (ID)	Control group (CG)
	Type (TP)	Attendance (A)	Other characteristics (OC)
Hartman et al. (2009)	F: 1/6W (educational sessions), 7/W (functionality maintenance EX) and 2/D (stretching and jumping EX)I: -TM: -TP: Education regarding possible motor problems resulting from chemotherapy, EX to maintain hand and leg function and stretching EX to maintain ankle dorsiflexion mobility and short-burst high-intensity EX to prevent reduction in BMD	V: -P: -ID: 2 yearsA: -	S: No (it was only supervised by their parents)PEP: HomeCG: Standard care for the CG included neither an initial session nor any prescheduled follow-up sessions with the hospital-based physiotherapistOC: Parents were supplied with an EX list, enabling them to select EX most appropriate for their child’s age and also to vary EX
Müller et al. (2014)	F: During hospital stays: preferably every second day. However, patients had the opportunity to work-out on a daily basis, except for the weekendsI: Moderate to vigorous (according to Borg’s ratings of perceived exertion of 13–16)TM: 15–45 MinTP: RT	V: 1–3 sets x 6–12 repsP: -ID: 6 MA: Patients participated in 34.5 ± 8 training sessions on average, corresponding to an adherence rate of 77%, based on the recommendation of training every other day	S: YesPEP: -CG: Received standard physiotherapeutic treatment based on their disability and as prescribed by the attending physician daily on workdays and included mobilization techniques of 20–30 Min durationOC: All patients received the same standard physiotherapeutic treatment than the CG. Additionally, sports games like football, basketball or table tennis were offered especially for younger children who could hardly be encouraged for the structured workouts
Cox et al. (2018)	F: 2/W (1st W—4th W), 1/W (5th W—8th W) and 1/M (9th W—135th W)I: -TM: -TP: Supporting motivation sessions about relatedness, competency, and autonomy	V: -P: NoID: 2.5 yearsA: There were no differences between the groups relative to APN (*p* = 0.12) missed appointments (intervention, missed APN visits, mean = 4.39, SD = 5.41; usual care, missed APN visits, mean = 2.49, SD = 3.60	S: No (it could have been supervised by their parents)PEP: HomeCG: Usual-care attention control (advanced practice nurse inquired in a neutral manner on the same schedule as for the intervention group)OC: It was emphasized the volitional nature of participation in the program and avoided coercive language)
Waked and Albenasy (2018)	F: 2/W (1st-6th M), 1/W (7st-12th M)	V: -	S: Yes
	I: Light to moderate (according to Borg’s ratings of perceived exertion of 3–6 out of 10)	P: Progression of EX for each patient depended on patient tolerance	PEP: -
	TM: 30–45 Min	ID: 12 M	CG: Each patient in CG was advised to be active as much as possible
	TP: Mixed-modality EX program: 1) AE such as walking or stationary cycling 2) RT using resistance bands 3) Flexibility training such as static stretching	A: -	OC: Necessary written instructions and tools such as resistance bands for prescribed EX were given to each child
Dubnov-Raz et al. (2015)	F: 3/W	V: -	S: No
	I: Moderate	P: -	PEP: Go-Active gym chain in Israel
	TM: 55–60 MinTP: Strength and endurance EX using bands, balls, games, free-weights and various EX machines in the gym	ID: 6 MA: -	CG: They were asked to continue with their usual lifestyle habitsOC: Adherence to the program was verified by telephone calls to the participants every two W and by periodic visits to the EG
Braam et al. (2018)	F: 2/W	V: -	S: Yes
	I: 66%–77% of HRpeak (1st W—4th W), 77%–90% of HRpeak (5th W—8th W) and 90%–100% of HRpeak (9th W—12th W)	P: The intensity of the physical EX training program gradually increased	PEP: Local physical therapy practice
	TM: 45 MinTP: AE and weight-bearing EX performed in a circuit training-setting with balls, hoops, and running activities	ID: 12 WA: The median adherence was 24 sessions (interquartile range (IQR): 20–24). 20 out of 30 children (67%) attended all physical EX training sessions within 12–16 W. 13% dropped-out mainly due to recurrence of the disease (7/9)	CG: Usual care according to local guidelines and preferencesOC: 10 children (33%) performed some of the EX at a lower heart rate than described
	F: At least 3/W (7th W—12th W)	V: -	S: No
	I: High intensity	P: No	PEP: Home
	TM: 11 Min	ID: 6 W (from 7th W)	CG: Usual care according to local guidelines and preferences
	TP: Weight-bearing EX	A: -	OC: N
	F: 1/W I: -TM: 60 Min TP: Psycho-education and cognitive-behavioral techniques including items on expression of feelings, self-perception and coping skills	V: -P: Yes	S: Yes PEP: - CG: Usual care according to local guidelines and preferences OC: After each individual session home EX on the topic of this specific session could be given to the patient if the psychologist considered it necessary
		ID: 12 W	
		A: The psychosocial training intervention was completed by 27 children (90%)	
Mogil et al. (2016)	F: Twice daily	V: -	S: No
	I: The mechanical signal (0.3 g at 32–37 Hz) produced a subtle, sinusoidal, vertical translation less than 100 μm *via* a linear electromagnetic actuator	P: -ID: 1 year	PEP: HomeCG: The placebo group stood on a device identical in appearance to the active platform. The placebo device emitted a 500-Hz audible hum but did not deliver the signal
	TM: 10 MinTP: Standing on an active platform	A: Median (interquartile range) values of 70.1% (35.4%–91.5%) in the intervention and 63.7% (33.3%–86.5%) in the placebo group (*p* = .40)	OC: Received calcium (800–1200 mg/d) and vitamin D supplements (cholecalciferol, 400 IU/d)
Elnaggar and Mohamed (2021)	F: 3/W I: Weight-bearing TM: 45 Min TP: Lower-body plyometric EX program	V: 10 lower-body Aqua-PLYO EX: 1st W—4th W: from 1 set x 4 reps to 3 sets x 10 reps 5th W—8th W: from 1 set x 15 reps to 3 sets x 15 reps 9th W—12th W: from 2 sets x 10 reps to 5 sets x 10 reps P: The training volume or intensity was increased as the W progressed in three blocks (specifically, every 4 W) ID: 12 W A: The median and interquartile range (IQR) of adherence-to-treatment was 91.67% (IQR 91.67% and 95.83%) in the Aqua-PLYO group and 95.83% (IQR 95.83% and 100%) in the CG	S: Yes PEP: 3 × 4 m water pool CG: Usual physical therapy OC: The water depth was waist-leveled, and the room and water temperature were regulated at 26°C–28°C and 30°C–31°C, respectively

Abbreviations: AE, aerobic exercise; EX, exercise; EG, exercise group; HRpeak, Heart Rate Peak; IQR, interquartile range; M, Month(s); D, day; Min, Minutes; “-”, not reported; RT, resistance training; SD, standard deviation; W, week.

### 3.4 Meta-analysis

**FIGURE 2:**
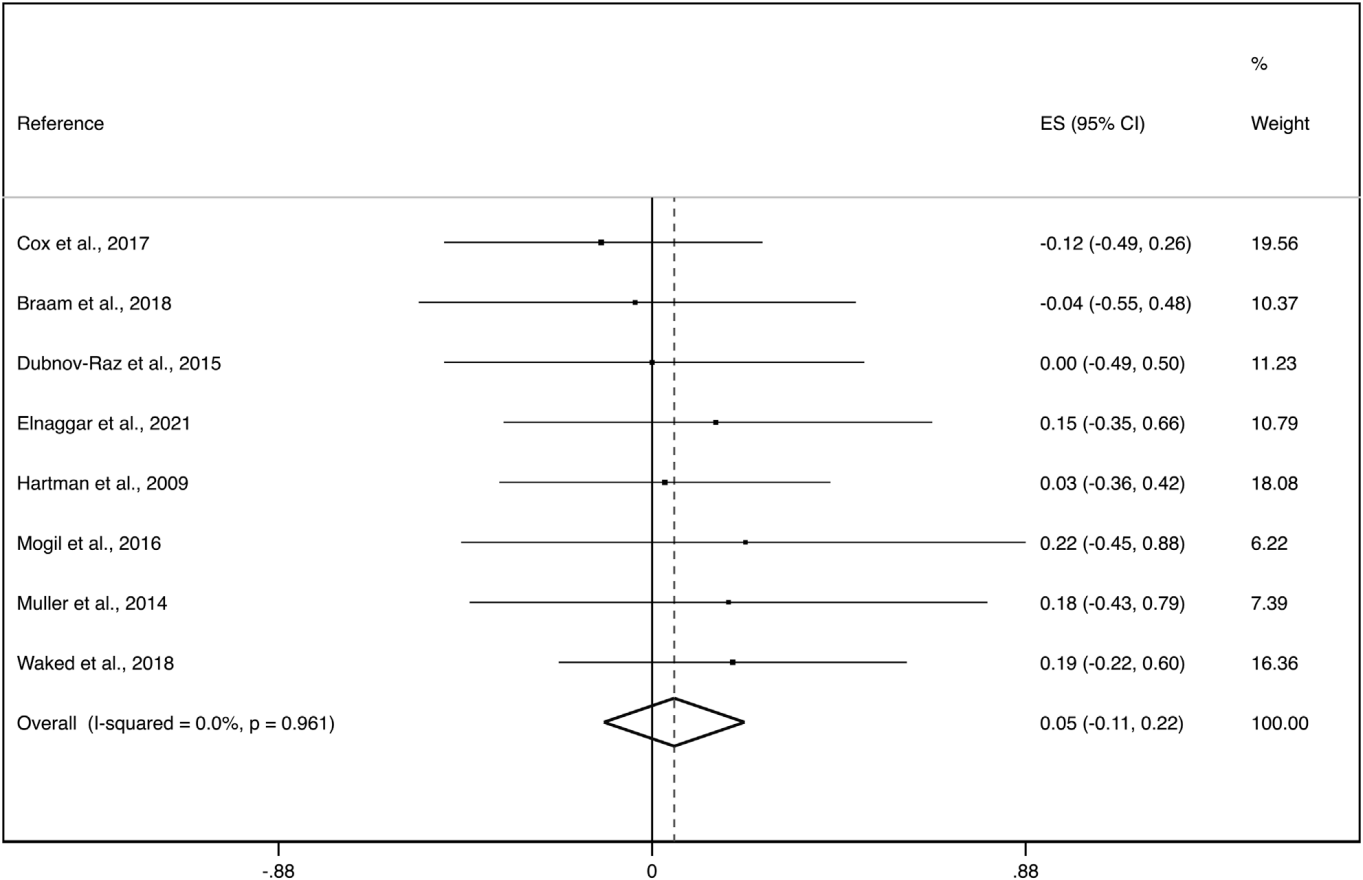
Forest plot of the effect size for the change in total aBMD. CI: confidence interval, ES: effect sizes.

The eight studies reporting aBMD changes after exercise interventions in paediatric cancer survivors during (n = 4) and after (n = 4) oncological treatment were included in this meta-analysis with a total of 341 participants. The pooled ES of exercise interventions showed no evidence of an effect on aBMD (ES = 0.05; 95%CI: −0.11, 0.22) with not important heterogeneity (I^2^ = 0.0%, *p* = 0.961; *τ2 = 0.000*). (Figure 2).

**FIGURE 3:**
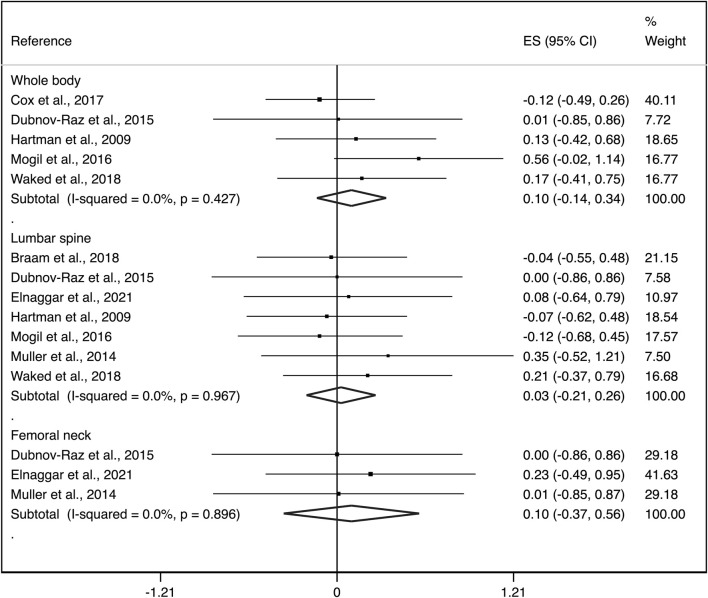
Forest plots of the effect size for the change in aBMD at the whole body, lumbar spine and femoral neck. CI: confidence interval, ES: effect sizes.

Exploratory subgroup analyses by aBMD region showed an ES of: i) 0.10 (95%CI: −0.14, 0.34) with not important heterogeneity (I^2^ = 0.0%, *p* = 0.427; τ*2 = 0.000*) for whole body, ii) 0.03 (95%CI: −0.21, 0.26) with not important heterogeneity (I^2^ = 0.0%, *p* = 0.967; τ*2 = 0.000*) for lumbar spine and, iii) 0.10 (95%CI: −0.37, 0.56) with no evidence of important heterogeneity (I^2^ = 0.0%, *p* = 0.896; τ*2 = 0.000*) for femoral neck (Figure 3).

**FIGURE 4:**
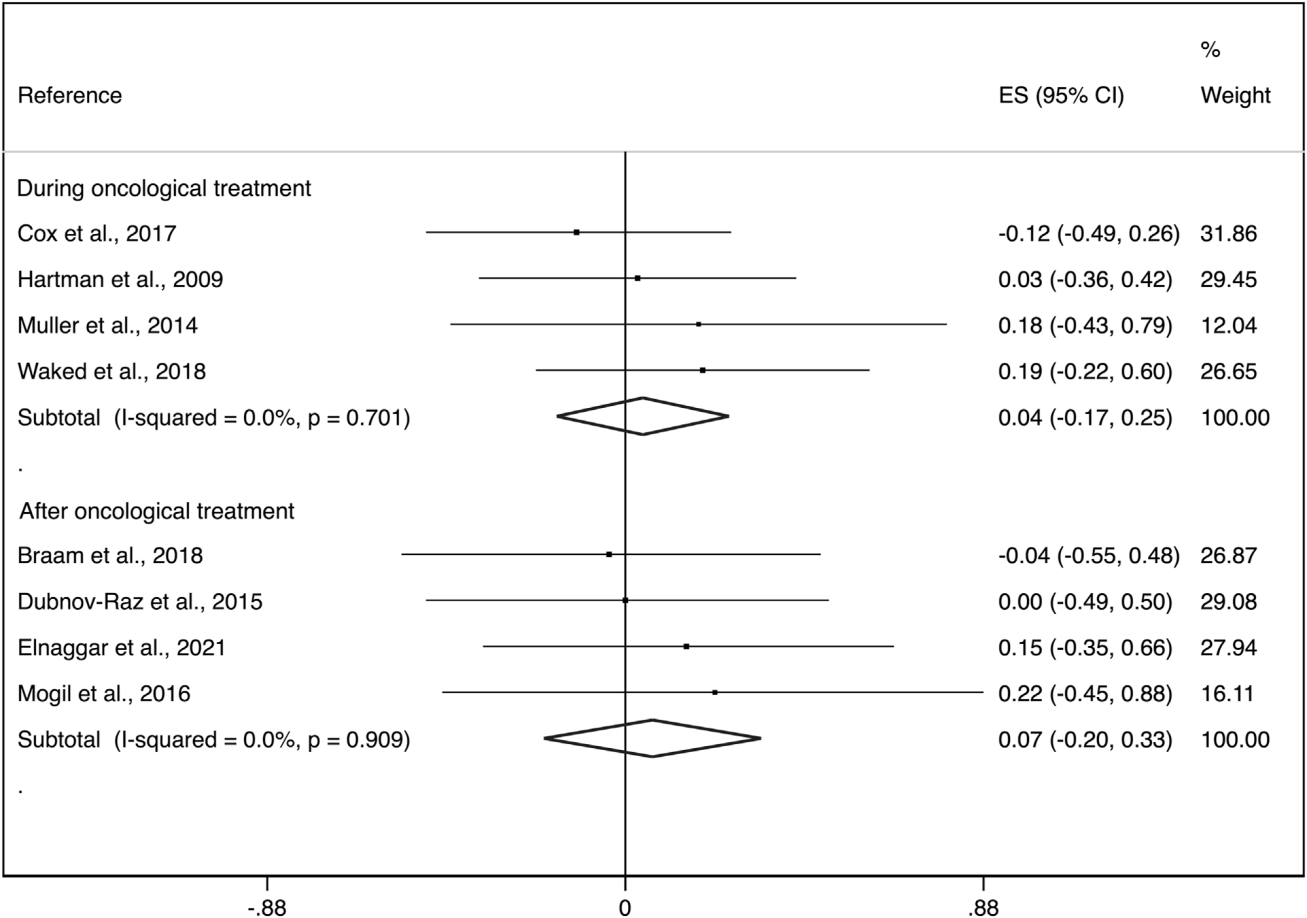
Forest plots of the effect size for the change in aBMD at the whole body, lumbar spine and femoral neck by groups (during cancer treatment and surviving patients). CI: confidence interval, ES: effect sizes.

Additionally, during the treatment phase the ES was: i) 0.04 (95%CI: −0.17, 0.25) with not important heterogeneity (I^2^ = 0.0%, *p* = 0.701; τ*2 = 0.000*) and after the treatment phase, ii) 0.07 (95%CI: −0.20, 0.33) with not important heterogeneity (I^2^ = 0.0%, *p* = 0.909; τ*2 = 0.000*) (Figure 4).

**FIGURE 5:**
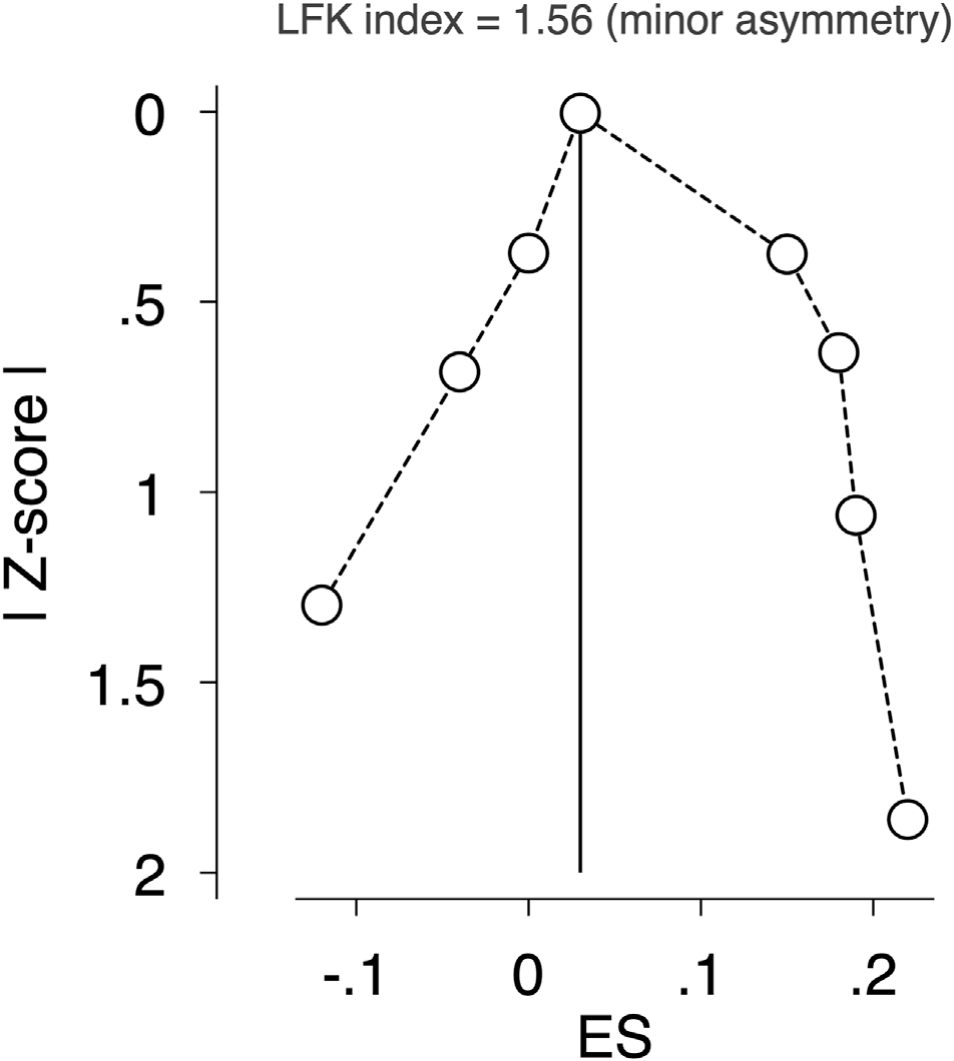
Assessment of potential publication bias by LFK index. Abbreviations: RCT, randomised controlled trial; WB, whole body; LS, lumbar spine; FN, femoral neck; aBMD, areal bone mineral density; vBMD, volumetric bone mineral density; BMAD, bone mineral apparent density; BMC, bone mineral content; SDS, standard deviation scores; NR, Not reported.

The pooled ES estimate for exercise interventions was not modified in aBMD when studies were removed from the analysis one at a time to examine the impact of individual studies. There was a minor asymmetry of small-study effects for exercise interventions, as evidenced by visual inspection of the Doi plot and LFK index (1.56) (Figure 5).

## 4 Discussion

The findings of the present systematic review and meta-analysis suggest that previous studies are inappropriate to illustrate any beneficial effect on improving bone parameters in children and adolescents during and after oncological treatment. Results should however be interpreted with caution due to the low number of the studies included and the low homogeneity of the intervention characteristics. To the best of our knowledge, this is the first systematic review and meta-analysis synthesising the evidence on the effect of exercise on bone health in children and adolescents during and after oncological treatment.

During oncological treatment, there are no studies showing a beneficial effect of exercise on bone parameters in children and adolescents. First and foremost, one of the most common side effects during oncological treatment is cancer-related fatigue (Lucía et al., 2003; Ng et al., 2005). This may be reflected by the poor adherence of participants to the exercise intervention as in the study of (Hartman et al., 2009), in which 36% of participants exercised less than once a week. This could have been an important barrier to achieve the required exercise intensity to effectively stimulate the bone and to obtain bone adaptations. As an example, previous research in healthy adolescents showed that those who did 28–32 min of vigorous physical activity per day had optimal aBMD at key regions within the hip (Gracia-Marco et al., 2011). Secondly, the prescribed exercise type might not be appropriate to bone adaptations in some studies. For instance, despite weight-bearing and impact exercises of high intensity significantly contribute bone development, this type of exercise was not chosen in the studies of (Müller et al., 2014) and Waked (Cox et al., 2018; Waked and Albenasy, 2018), and when included, the intensity required to modify bone parameters were not achievable as mentioned in the study of (Cox et al., 2018). The latter intervention was proven not to be feasible during the early oncological treatment phase owing to the child’s responses to the disease and the treatment. Interestingly, this exercise intervention was the longest (30 months) in comparison with the rest of studies. Finally, it is important to mention that half of the exercise interventions were unsupervised (Hartman et al., 2009; Cox et al., 2018), which does not concur with the International Pediatric Oncology Exercise Guidelines which recommend that a qualified exercise professionals should implement supervised exercise programmes throughout cancer continuum (Wurz et al., 2021).

To sum up, the most updated research in children and adolescents during oncological treatment suggests that there is no evidence of an effect of exercise at inducing meaningful bone adaptations. Overall, the potential cancer-related fatigue sequel, the selection of the inappropriate exercises to improve bone parameters (e.g., cycling, lack of weight-bearing impact exercises of high intensity, unsupervised exercise interventions) and the unachievable intensity of the interventions are important factors that have hindered the required stimulus in the bones. The use of behaviour change techniques (i.e., gamification) in long-lasting interventions with growing population is recommended (Muntaner-Mas et al., 2017; Sailer et al., 2017) and could have helped to increase the low adherence rate reported (Hartman et al., 2009; Cox et al., 2018).

Shortly after oncological treatment, there is no evidence of positive effects of exercise interventions aimed at improving bone parameters. One of the potential factors could be the short duration as half of the interventions lasted for only 3 months (Braam et al., 2018; Elnaggar and Mohamed, 2021). The bone remodelling process takes approximately 5 months and therefore, shorter interventions could not reflect true bone adaptations (Kenkre and Bassett, 2018). In addition, the type of exercise has not been the most appropriate to improve bone parameters in some cases (Dubnov-Raz et al., 2015) did not include weight-bearing impact exercises of high intensity yet participants reported to be mentally and physically healthier than those in previous studies during oncological treatment (Cox et al., 2018). Likewise, Elnaggar et al. (Elnaggar and Mohamed, 2021) included lower-body plyometric exercises in a swimming pool, that is, in a microgravity environment, which is not effective at increasing bone parameters (Gómez-Bruton et al., 2013). Nevertheless, (Mogil et al., 2016) implemented an intervention including standing on an active vibration platform emitting low-magnitude high-frequency mechanical stimulation, considered a type of weight-bearing physical activity as it requires muscles and bones to work against gravity (Cardinale and Bosco, 2003; Cardinale and Wakeling, 2005). From the included studies, the latter was the only intervention that observed a borderline significant increase in total body aBMD (*p* = 0.05). The timing of the intervention (i.e., after oncological treatment), the frequency (twice per day) and adequate intervention duration (1 year) could explain the findings. However, their intervention type was clearly ineffective at increasing lumbar spine aBMD outcomes. As stated by the authors, this might have been caused by the potential loss of vibratory energy as the signal travelled from the distal lower extremity to the trunk. This agrees with a recent systematic review and meta-analysis in children and adolescents with motor disabilities that found no pooled effect of similar interventions on lumbar spine aBMD (Li et al., 2022). Lastly, some studies did not exclude participants receiving growth hormone, corticosteroids or bisphosphonates (Dubnov-Raz et al., 2015), or even included participants during the remaining oncological treatment period (Braam et al., 2018), which might have affected the results.

In conclusion, there is no evidence of an effect of exercise interventions conducted after oncological treatment at increasing bone parameters in children and adolescents. There are several reasons that may explain this lack of effect: the short duration of the interventions, the type of the exercises (i.e., lack of weight-bearing exercises or in a microgravity environment) and inclusion of participants undergoing maintenance treatment that affects bone parameters.

Remarkably, the exercise interventions were not delivered by exercise professionals in 75% of the included studies. This sets a potential barrier and limitation for the intervention to succeed. There is a need of exercise professionals with a high qualification and robust background in exercise oncology. Similar thoughts have been shared by (Adams et al., 2021) who stated that oncologic healthcare providers working in cancer care system did not feel confident when prescribing exercise and therefore, they should not be responsible for prescribing it. According to the International Pediatric Oncology Exercise Guidelines, qualified exercise professionals should be part of standard care and therefore should facilitate programme implementation and uptake throughout the cancer continuum (Wurz et al., 2021).

The present systematic review and meta-analysis has several limitations. The main limitation is the availability of published studies and well-designed RCTs aiming at investigating bone changes in children and adolescents diagnosed with cancer. Additionally, the data reported were exclusively taken from the manuscripts included in this work and not from the clinical trials registries. In most of the cases, the interventions were not designed to meet the aim of improving bone health. Thus, these findings should be viewed with caution. Nevertheless, it shows the current evidence on exercise paediatric oncology and bone health and should be viewed as a starting point for researchers to think of the best approach for designing their exercise interventions. To date, only two systematic reviews and meta-analyses have been conducted with the same purpose in adult cancer patients during and after oncological treatment with promising positive results (Rose et al., 2022; Singh and Toohey, 2022).

## 5 Conclusion

Our systematic review and meta-analysis indicate that the exercise interventions were inappropriate and therefore, ineffective to illustrate any beneficial effect on bone of children and adolescents with cancer during and after oncological treatment. Several limitations in the design of the interventions have been identified. There is a need of implementing well-designed exercise RCTs specifically focused on improving bone health in children and adolescents diagnosed with cancer due its scientific and clinical importance. Early intervention strategies to optimize bone health through effective tailoring of osteogenic exercise programmes are of vital importance.

## Data Availability

The raw data supporting the conclusions of this article will be made available by the authors, without undue reservation.

## References

[B1] Adams J. Rauw J. Weller S. Campbell K. L. Pollock P. Goulart J. (2021). Physical activity recommendations for cancer survivors living with bony metastases: Views of oncologic healthcare providers. J. Cancer Surviv 15 (3), 414–417. 10.1007/s11764-021-00999-8 33604871

[B2] Ardern C. L. Büttner F. Andrade R. Weir A. Ashe M. C. Holden S. (2022). Implementing the 27 PRISMA 2020 statement items for systematic reviews in the sport and exercise medicine, musculoskeletal rehabilitation and sports science fields: The PERSiST (implementing prisma in exercise, rehabilitation, sport medicine and SporTs science) guidance. Br. J. Sports Med. 56, 175–195. [Internet]. 10.1136/bjsports-2021-103987 34625401 PMC8862073

[B3] Bailey D. A. McKay H. A. Mirwald R. L. Crocker P. R. E. Faulkner R. A. (1999). A six-year longitudinal study of the relationship of physical activity to bone mineral accrual in growing children: The University of saskatchewan bone mineral accrual study. J. Bone Min. Res. 14 (10), 1672–1679. 10.1359/jbmr.1999.14.10.1672 10491214

[B4] Bonjour J. P. Chevalley T. Rizzoli R. Ferrari S. (2007). Gene-environment interactions in the skeletal response to nutrition and exercise during growth. Med. Sport Sci. 51, 64–80. 10.1159/000103005 17505120

[B5] Braam K. I. van Dijk-Lokkart E. M. Kaspers G. J. L. Takken T. Huisman J. Buffart L. M. (2018). Effects of a combined physical and psychosocial training for children with cancer: A randomized controlled trial. BMC Cancer 18 (1), 1289. [Internet]. 10.1186/s12885-018-5181-0 30587148 PMC6307314

[B6] Campbell K. L. Winters-Stone K. M. Wiskemann J. May A. M. Schwartz A. L. Courneya K. S. (2019). Exercise guidelines for cancer survivors: Consensus statement from international multidisciplinary roundtable. Med. Sci. Sports Exerc 51 (11), 2375–2390. 10.1249/MSS.0000000000002116 31626055 PMC8576825

[B7] Cardinale M. Bosco C. (2003). The use of vibration as an exercise intervention. Exerc Sport Sci. Rev. 31 (1), 3–7. 10.1097/00003677-200301000-00002 12562163

[B8] Cardinale M. Wakeling J. (2005). Whole body vibration exercise: Are vibrations good for you? Br. J. Sports Med. 39, 585–589. [Internet]. 10.1136/bjsm.2005.016857 16118292 PMC1725325

[B9] Cox C. L. Zhu L. Kaste S. C. Srivastava K. Barnes L. Nathan P. C. (2018). Modifying bone mineral density, physical function, and quality of life in children with acute lymphoblastic leukemia. Pediatr. Blood Cancer 65 (4), 269299–e26938. [Internet]. 10.1002/pbc.26929 PMC5821547 29286560

[B10] Davies J. H. Evans B. A. J. Gregory J. W. (2005). Bone mass acquisition in healthy children. Arch. Dis. Child. 90 (4), 373–378. [Internet][cited 2022 May 11];. 10.1136/adc.2004.053553 15781927 PMC1720329

[B11] Dubnov-Raz G. Azar M. Reuveny R. Katz U. Weintraub M. Constantini N. W. (2015). Changes in fitness are associated with changes in body composition and bone health in children after cancer. Acta Paediatr. 104 (10), 1055–1061. [Internet]. 10.1111/apa.13052 26011285

[B12] Elnaggar R. K. Mohamed R. R. (2021). Aqua-plyometric exercises: Potential implications for bone mineral density, functional capacity, and quality of life in survivors of childhood acute lymphoblastic leukemia. Semin. Oncol. Nurs. 37 (6), 151225. [Internet]. 10.1016/j.soncn.2021.151225 34753640

[B13] Furuya-Kanamori L. Barendregt J. J. Doi S. A. R. (2018). A new improved graphical and quantitative method for detecting bias in meta-analysis. Int. J. Evid. Based Healthc. 16 (4), 195–203. [Internet][cited 2023 Jan 17];. 10.1097/XEB.0000000000000141 29621038

[B14] Gómez-Bruton A. Gónzalez-Agüero A. Gómez-Cabello A. Casajús J. A. Vicente-Rodríguez G. (2013). Is bone tissue really affected by swimming? A systematic review. PLoS One 8 (8), e70119. 10.1371/journal.pone.0070119 23950908 PMC3737199

[B15] Gómez-Bruton A. Matute-Llorente Á González-Agüero A. Casajús J. A. Vicente-Rodríguez G. (2017). Plyometric exercise and bone health in children and adolescents: A systematic review. World J. Pediatr. 13 (2), 112–121. 10.1007/s12519-016-0076-0 28101776

[B16] Gracia-Marco L. Moreno L. A. Ortega F. B. Len F. Sioen I. Kafatos A. (2011). Levels of physical activity that predict optimal bone mass in adolescents: The HELENA study. Am. J. Prev. Med. 40, 599–607. [Internet]. 10.1016/j.amepre.2011.03.001 21565650

[B17] Harel Z. Gold M. Cromer B. Bruner A. Stager M. Bachrach L. (2007). Bone mineral density in postmenarchal adolescent girls in the United States: Associated biopsychosocial variables and bone turnover markers. J. Adolesc. Health 40 (1), 44–53. 10.1016/j.jadohealth.2006.08.013 17185205

[B18] Hartman A. te Winkel M. L. van Beek R. D. de Muinck Keizer-Schrama S. M. P. F. Kemper H. C. G. Hop W. C. J. (2009). A randomized trial investigating an exercise program to prevent reduction of bone mineral density and impairment of motor performance during treatment for childhood acute lymphoblastic leukemia. Pediatr. Blood Cancer 53 (1)–6471. [Internet]. 10.1002/pbc.21942 19283791

[B19] Higgins J. P. T. Thomas J. Chandler J. Cumpston M. Li T. Page M. J. (2022). Cochrane Handbook for systematic reviews of interventions version 6.3. (updated February 2022). [Internet]. Cochrane Available at: www.training.cochrane.org/handbook . 10.1002/14651858.ED000142 PMC10284251 31643080

[B20] Higgins J. P. T. Thompson S. G. (2002). Quantifying heterogeneity in a meta-analysis. Stat. Med. 21 (11), 1539–1558. 10.1002/sim.1186 12111919

[B21] Kelly P. M. Pottenger E. (2022). Bone health issues in the pediatric oncology patient. Semin. Oncol. Nurs. 38 (2), 151275. 10.1016/j.soncn.2022.151275 35491332 PMC9516783

[B22] Kenkre J. S. Bassett J. (2018). The bone remodelling cycle. Ann. Clin. Biochem. 55 (3), 308–327. 10.1177/0004563218759371 29368538

[B23] Li S. Yu W. Li W. Wang J. Gao L. Li S. (2022). The impact of whole-body vibration training on bone minerals and lean mass in children and adolescents with motor disabilities: A systematic review and meta-analysis. Child. (Basel) 9 (2). [Internet]. 10.3390/children9020266 PMC8870738 35204986

[B24] Lucía A. Earnest C. Pérez M. (2003). Cancer-related fatigue: Can exercise physiology assist oncologists? Lancet Oncol. 4 (10), 616–625. [Internet]. 10.1016/s1470-2045(03)01221-x 14554239

[B25] Marcucci G. Beltrami G. Tamburini A. Body J. J. Confavreux C. B. Hadji P. (2019). Bone health in childhood cancer: Review of the literature and recommendations for the management of bone health in childhood cancer survivors. Ann. Oncol. 30 (6), 908–920. [Internet]. 10.1093/annonc/mdz120 31111878

[B26] Miller K. D. Fidler-Benaoudia M. Keegan T. H. Hipp H. S. Jemal A. Siegel R. L. (2020). Cancer statistics for adolescents and young adults. CA Cancer J. Clin. 70 (6), 443–459. [Internet]. 2020 Nov [cited 2022 Sep 13];. 10.3322/caac.21637 32940362

[B27] Mogil R. J. Kaste S. C. Ferry R. J. Hudson M. M. Mulrooney D. A. Howell C. R. (2016). Effect of low-magnitude, high-frequency mechanical stimulation on BMD among young childhood cancer survivors: A randomized clinical trial. JAMA Oncol. 2 (7), 908–914. [Internet]. 10.1001/jamaoncol.2015.6557 26967465 PMC4945422

[B28] Morales J. S. Valenzuela P. L. Velázquez-Díaz D. Castillo-García A. Jiménez-Pavón D. Lucia A. (2021). Exercise and childhood cancer-A historical review. Cancers (Basel) 14 (1), 82. 10.3390/cancers14010082 35008246 PMC8750946

[B29] Müller C. Winter C. Boos J. Gosheger G. Hardes J. Vieth V. (2014). Effects of an exercise intervention on bone mass in pediatric bone tumor patients. Int. J. Sports Med. 35, 696–703. [Internet]. 10.1055/s-0033-1358475 24408763

[B30] Muntaner-Mas A. Vidal-Conti J. Borràs P. A. Ortega F. B. Palou P. (2017). Effects of a Whatsapp-delivered physical activity intervention to enhance health-related physical fitness components and cardiovascular disease risk factors in older adults. J. Sports Med. Phys. Fit. 57 (1–2), 90–102. 10.23736/S0022-4707.16.05918-1 26364690

[B31] Ng A. K. Li S. Recklitis C. Neuberg D. Chakrabarti S. Silver B. (2005). A comparison between long-term survivors of Hodgkin’s disease and their siblings on fatigue level and factors predicting for increased fatigue. Ann. Oncol. 16 (12), 1949–1955. [Internet]. 10.1093/annonc/mdi407 16227316

[B32] Page M. J. McKenzie J. E. Bossuyt P. M. Boutron I. Hoffmann T. C. Mulrow C. D. (2021). The PRISMA 2020 statement: An updated guideline for reporting systematic reviews. BMJ 372, n71. 10.1136/bmj.n71 33782057 PMC8005924

[B33] Rizzoli R. Bianchi M. L. Garabédian M. McKay H. A. Moreno L. A. (2010). Maximizing bone mineral mass gain during growth for the prevention of fractures in the adolescents and the elderly. Bone 46 (2), 294–305. 10.1016/j.bone.2009.10.005 19840876

[B34] Rodd C. Kirouac N. Orkin J. Grimes R. (2022). Evaluating and optimizing bone health in children with chronic health conditions. Paediatr. Child. Health 27 (4), 232–236. [Internet]. 10.1093/pch/pxac036 PMC9291353 35859678

[B35] Rose G. L. Skinner T. L. Keating S. E. Friedrich N. K. Bolam K. A. (2022). The effects of exercise on the bone health of people with cancer: A systematic review and meta-analysis. Osteoporos. Int. 33 (2), 327–338. 10.1007/s00198-021-06131-x 34532766

[B36] Sailer M. Hense J. U. Mayr S. K. Mandl H. (2017). How gamification motivates: An experimental study of the effects of specific game design elements on psychological need satisfaction. Comput. Hum. Behav. 69, 371–380. 10.1016/j.chb.2016.12.033

[B37] Siegel R. L. Miller K. D. Sandeep N. Mbbs W. Ahmedin J. (2023). Cancer statistics. CA Cancer J. Clin. 73 (1), 17–48. [Internet]2023 Jan 1 [cited 2023 Jan 16];. 10.3322/caac.21763 36633525

[B38] Singh B. Toohey K. (2022). The effect of exercise for improving bone health in cancer survivors — a systematic review and meta-analysis. J. Sci. Med. Sport 25 (1), 31–40. 10.1016/j.jsams.2021.08.008 34465518

[B39] Sterne J. A. C. Savović J. Page M. J. Elbers R. G. Blencowe N. S. Boutron I. (2019). RoB 2: A revised tool for assessing risk of bias in randomized trials. BMJ 366. [Internet]. 10.1136/bmj.l4898 31462531

[B40] Stettler C. Allemann S. Wandel S. Kastrati A. Morice M. C. Schömig A. (2008). Drug eluting and bare metal stents in people with and without diabetes: Collaborative network meta-analysis. BMJ 337, a1331. [Internet][cited 2023 Jan 17];. 10.1136/bmj.a1331 18757996 PMC2527175

[B41] Trama A. Botta L. Foschi R. Ferrari A. Stiller C. Desandes E. (2016). Survival of European adolescents and young adults diagnosed with cancer in 2000-07: Population-based data from EUROCARE-5. Lancet Oncol. 17 (7), 896–906. [Internet][cited 2022 Jul 25]. 10.1016/S1470-2045(16)00162-5 27237614

[B42] Tufanaru C. Munn Z. Aromataris E. Campbell J. Hopp L. (2017). “Chapter 3: Systematic reviews of effectiveness,” in Joanna Briggs Institute reviewer’s manual. Editors Aromataris E. Munn Z. (The Joanna Briggs Institute). [Internet]Available at: https://reviewersmanual.joannabriggs.org/.

[B43] Ubago-Guisado E. Martinez-Rodriguez A. Gallardo L. Sánchez-Sánchez J. (2016). Bone mass in girls according to their BMI, VO2 max, hours and years of practice. Eur. J. Sport Sci. 16 (8), 1176–1186. 10.1080/17461391.2016.1168484 27050621

[B44] Ubago-Guisado E. Vlachopoulos D. Barker A. R. Christoffersen T. Metcalf B. Gracia-Marco L. (2019). Effect of maturational timing on bone health in male adolescent athletes engaged in different sports: The PRO-BONE study. J. Sci. Med. Sport 22 (3), 253–258. [Internet]. 10.1016/j.jsams.2018.08.009 30146475

[B45] Vlachopoulos D. Barker A. R. Ubago-Guisado E. Williams C. A. Gracia-Marco L. (2018). A 9-month jumping intervention to improve bone geometry in adolescent male athletes. Med. Sci. Sports Exerc 50 (12), 2544–2554. 10.1249/MSS.0000000000001719 30067592

[B46] Vlachopoulos D. Barker A. R. Ubago-Guisado E. Williams C. A. Gracia-Marco L. (2018). The effect of a high-impact jumping intervention on bone mass, bone stiffness and fitness parameters in adolescent athletes. Arch. Osteoporos. 13 (1), 128. 10.1007/s11657-018-0543-4 30446875 PMC6244891

[B47] Waked I. Albenasy K. (2018). Bone mineral density, lean body mass and bone biomarkers following physical exercise in children with acute lymphoblastic leukemia undergoing chemotherapy. IJBC 10 (3), 69–75.

[B48] Weaver C. M. Gordon C. M. Janz K. F. Kalkwarf H. J. Lappe J. M. Lewis R. (2016). The national osteoporosis foundation’s position statement on peak bone mass development and lifestyle factors: A systematic review and implementation recommendations. Osteoporos. Int. 27 (4), 1281–1386. 10.1007/s00198-015-3440-3 26856587 PMC4791473

[B49] Wilson C. L. Ness K. K. (2013). Bone mineral density deficits and fractures in survivors of childhood cancer. Curr. Osteoporos. Rep. 11 (4), 329–337. 10.1007/s11914-013-0165-0 24043370 PMC4260527

[B50] Wurz A. McLaughlin E. Lategan C. Chamorro Viña C. Grimshaw S. L. Hamari L. (2021). The international pediatric oncology exercise guidelines (iPOEG). Transl. Behav. Med. 11 (10), 1915–1922. 10.1093/tbm/ibab028 34037786 PMC8604278

