# Supplementary material for: Effect of exercise on bone health in children and adolescents with cancer during and after oncological treatment: A systematic review and meta-analysis
Source: Front Physiol. 2023 Mar 14;14:1088740. doi: 10.3389/fphys.2023.1088740 (PMC10081564; doi:10.3389/fphys.2023.1088740)
Supplement: Supplementary file 2 [file Table2.DOCX]

| **Supplementary Appendix S2.** Search terms used in databases. |
| --- |
| MEDLINE (via PubMed) |
| (exercis*[Title/Abstract] OR move*[Title/Abstract] OR moving[Title/Abstract] OR sport*[Title/Abstract] OR train*[Title/Abstract] OR “physical activity”[Title/Abstract] OR weightbear*[Title/Abstract] OR “high impact”[Title/Abstract] OR running[Title/Abstract] OR walk*[Title/Abstract] OR strength*[Title/Abstract] OR “physical fitness”[Title/Abstract] OR step*[Title/Abstract] OR gymnastic[Title/Abstract] OR balance[Title/Abstract]) AND (bone[Mesh] OR bone[Title/Abstract]) AND (cancer[Title/Abstract] OR oncology[Mesh] OR onco*[Title/Abstract] OR myelo*[Title/Abstract] OR leukaemia[Title/Abstract] OR leukemia[Title/Abstract] OR neoplasm*[Title/Abstract] OR lympho*[Title/Abstract] OR carcinoma[Title/Abstract] OR tumor[Title/Abstract] OR tumour[Title/Abstract] OR sarcoma[Title/Abstract]) AND (child*[Title/Abstract] OR adolescen*[Title/Abstract] OR young*[Title/Abstract] OR boy*[Title/Abstract] OR girl*[Title/Abstract] OR pediatric*[Title/Abstract] OR paediatric*[Title/Abstract]) AND (trial*[Title/Abstract] OR random[Title/Abstract] OR intervention*[Title/Abstract] OR program*[Title/Abstract] OR rehabilitation[Title/Abstract]) |
| Web of Science |
| AB=(( exercis* OR move* OR moving OR sport* OR train* OR “physical activity” OR weightbear* OR “high impact” OR running OR walk* OR strength* OR “physical fitness” OR step* OR gymnastic OR balance) AND ( bone ) AND ( cancer OR onco* OR myelo* OR leukaemia OR leukemia OR neoplasm* OR lympho* OR carcinoma OR tumor OR tumour OR sarcoma ) AND ( child* OR adolescen* OR young* OR boy* OR girl* OR pediatric* OR paediatric* ) AND ( trial* OR random OR intervention* OR program* OR rehabilitation )) |
| Scopus |
| (TITLE-ABS(exercis*) OR TITLE-ABS(move*) OR TITLE-ABS(moving) OR TITLE-ABS(sport*) OR TITLE-ABS(train*) OR TITLE-ABS(“physical activity”) OR TITLE-ABS(weightbear*) OR TITLE-ABS(“high impact”) OR TITLE-ABS(running) OR TITLE-ABS(walk*) OR TITLE-ABS(strength*) OR TITLE-ABS(“physical fitness”) OR TITLE-ABS(step*) OR TITLE-ABS(gymnastic) OR TITLE-ABS(balance)) AND (TITLE-ABS(bone)) AND (TITLE-ABS(cancer) OR TITLE-ABS(onco*) OR TITLE-ABS(myelo*) OR TITLE-ABS(leukaemia) OR TITLE-ABS(leukemia) OR TITLE-ABS(neoplasm*) OR TITLE-ABS(lympho*) OR TITLE-ABS(carcinoma) OR TITLE-ABS(tumor) OR TITLE-ABS(tumour) OR TITLE-ABS(sarcoma)) AND (TITLE-ABS(child*) OR TITLE-ABS(adolescen*) OR TITLE-ABS(young*) OR TITLE-ABS(boy*) OR TITLE-ABS(girl*) OR TITLE-ABS(pediatric*) OR TITLE-ABS(paediatric*)) AND (TITLE-ABS(trial*) OR TITLE-ABS(random) OR TITLE-ABS(intervention*) OR TITLE-ABS(program*) OR TITLE-ABS(rehabilitation)) |
