# Supplementary material for: Effect of exercise on bone health in children and adolescents with cancer during and after oncological treatment: A systematic review and meta-analysis
Source: Front Physiol. 2023 Mar 14;14:1088740. doi: 10.3389/fphys.2023.1088740 (PMC10081564; doi:10.3389/fphys.2023.1088740)

**Supplementary Appendix S3.** Quality assessment of included articled for Randomized Controlled Trials.


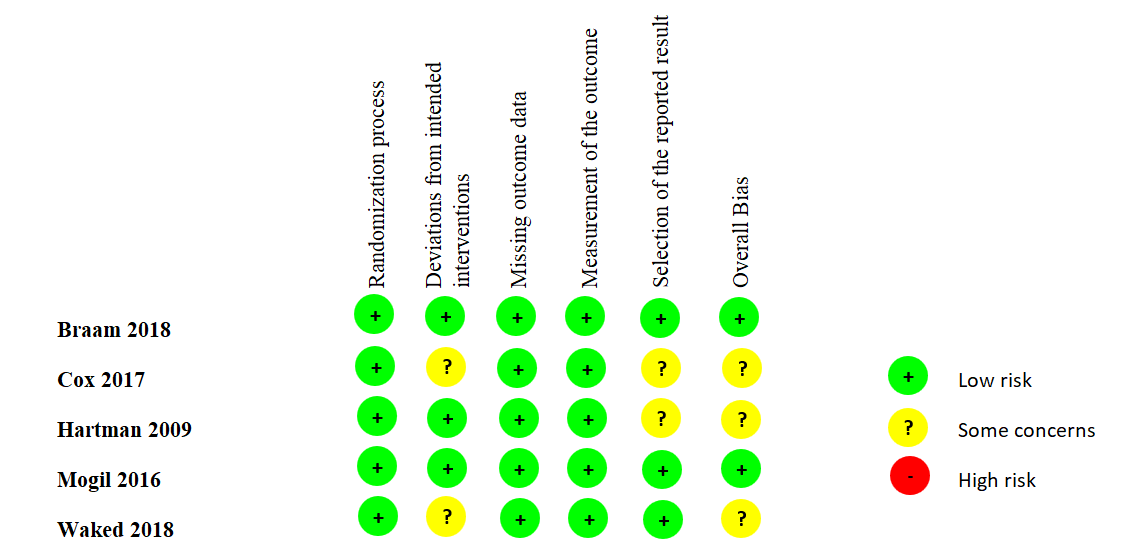

Supplement: Supplementary file 3 [file Table3.DOCX]
