# Supplementary material for: Effect of exercise on bone health in children and adolescents with cancer during and after oncological treatment: A systematic review and meta-analysis
Source: Front Physiol. 2023 Mar 14;14:1088740. doi: 10.3389/fphys.2023.1088740 (PMC10081564; doi:10.3389/fphys.2023.1088740)
Supplement: Supplementary file 4 [file Table4.DOCX]

| **Supplementary Appendix S4.** Quality assessment of included articles for Quasi-Experimental Studies. | | | | | | | | | | |
| --- | --- | --- | --- | --- | --- | --- | --- | --- | --- | --- |
| **Study** | **Item 1** | **Item 2** | **Item 3** | **Item 4** | **Item 5** | **Item 6** | **Item 7** | **Item 8** | **Item 9** | **Quality Category** |
| Dubnov-Raz 2015 | ✓ | ✓ | 🗶 | ✓ | ✓ | ✓ | ✓ | ✓ | 🗶 | High |
| Elnaggar 2021 | ✓ | ✓ | ✓ | ✓ | ✓ | ✓ | ✓ | ✓ | ✓ | High |
| Müller 2014 | ✓ | 🗶 | 🗶 | ✓ | ✓ | ✓ | ✓ | ✓ | 🗶 | Medium |
| **Criterion Score %** | 100 | 66.67 | 33.33 | 100 | 100 | 100 | 100 | 100 | 33.33 |  |
| Note that the criterion score is calculated by dividing the number of studies meeting one criterion by the total number of studies. ✓: meet the methodological quality criterion; 🗶: not meet the methodological quality criterion.; ?: unclear; N/A: not applicable. | | | | | | | | | | |
